# Combined double chambered right ventricle, tricuspid valve dysplasia, ventricular septal defect, and subaortic stenosis in a dog

**DOI:** 10.1186/s12917-017-1275-1

**Published:** 2017-11-29

**Authors:** Iuliu Scurtu, Flaviu Tabaran, Mircea Mircean, Gavril Giurgiu, Andras Nagy, Cornel Catoi, Dan G. Ohad

**Affiliations:** 1 Universitatea de Stiinte Agricole si Medicina Veterinara din Cluj-Napoca, Calea Manastur 3-5, 400372 Cluj-Napoca, Romania; 2 The Koret School of Veterinary Medicine, Robert H. Smith Faculty of Agriculture Food and Environment, Hebrew University of Jerusalem, P.O. Box 12, 76100 Rehovot, Israel

**Keywords:** Canine, Congenital heart disease, Echocardiography

## Abstract

**Background:**

Double chambered right ventricle (DCRV) is a congenital heart anomaly where the right ventricle is divided into two chambers. We describe, for the first time, an unusual combination of DCRV combined with some other congenital heart defects.

**Case presentation:**

A 1.2-year-old Golden Retriever was presented with lethargy, exercise intolerance and ascites. Physical examination revealed an irregularly irregular pulse and a grade V/VI, systolic, right cranial murmur. Electrocardiography revealed widened and splintered QRS complexes with a right bundle-branch block pattern. Radiography demonstrated right-sided cardiomegaly. Two-dimensional echocardiography identified a DCRV with tricuspid valve dysplasia. The patient died despite abdominocentesis and 4 days of oral pharmacotherapy, and necropsy revealed an anomalous fibromuscular structure that divided the right ventricle into two compartments. Another finding was tricuspid valve dysplasia with hypoplasia of the posterior and septal leaflets. The anterior leaflet was prominent, being part of the anomalous structure that divided the right ventricle. Necropsy also identified a perimembranous ventricular septal defect and mild subaortic stenosis. Histopathological examination of the fibromuscular band that separated the right ventricle identified longitudinally oriented layers of dense fibrous connective tissue and myocardial cells arranged in a plexiform pattern. The muscular component was well represented at the ventral area of the fibromuscular band, and was absent in the central zone. Superficially, the endocardium presented areas of nodular hyperplasia covering mainly the fibrous part of the abnormal structure. The nodules were sharply demarcated and were composed by loosely arranged connective tissue with myxoid appearance, covered by discrete hyperplastic endocardium.

**Conclusions:**

Concomitant cardiac malformations involving DCRV, tricuspid valve dysplasia, perimembranous ventricular septal defect and mild subaortic stenosis have not been previously described in veterinary medicine, and are reported here for the first time. Moreover, this is the first report of a canine patient with tricuspid valve dysplasia (TVD) and DCRV where the anterior leaflet is part of an anomalous structure dividing the right ventricle (RV) into two separate compartments.

## Background

A double chambered right ventricle (DCRV) is a rarely diagnosed congenital heart anomaly, where a fibromuscular band separates the right ventricle (RV) into two chambers [1, 2]. This condition has been described in dogs, human beings [3–5], cats [6], and alpacas [7]. The dog breeds most frequently affected by this anomaly are large, such as Golden Retriever [2, 8] and Boxer [9]. A case of DCRV was also reported in a Pug [10]. One study reported no sex predisposition [10] and another suggested that males were overrepresented [8]. There are two types of DCRV reported in human beings: a low (oblique) type [11] and a high (horizontal) type [5, 11]. In both types, an anomalous development of septoparietal trabeculation has been reported [11], but there is currently no consensus regarding the exact pathogenesis behind the obstructing muscle bundles [12].

In a veterinary study, DCRV represented 1.2% (14/1132) of identified congenital heart pathologies [13]. In human beings, DCRV evolves as an isolated anomaly in only exceptionally rare instances [3]. Similarly, in dogs, most frequently, DCRV can be associated with ventricular septal defect (VSD), atrial septal defect (ASD), tricuspid valve dysplasia (TVD), pulmonic stenosis (PS), subaortic stenosis (SAS) or patent ductus arteriosus (PDA) [2, 8, 10].

In human beings, a genetic predisposition has been postulated [14]. There were also cases reported with Down’s syndrome and DCRV, and a possible relationship between these two conditions was proposed [15]. It was also suggested that there were multiple embryological and postnatal causes of DCRV, as cardiac morphogenesis is an extremely complex and interconnected process [5]. Another theory considered DCRV as an acquired cardiac defect of postnatal development, as the coexistence of discrete aortic stenosis might imply a genetic predisposition toward cellular proliferation [16].

In dogs, due to the lack of well-documented data, epidemiological and morphological features are not clearly understood [8]. The most frequently encountered presenting complaints include cough, exercise intolerance, cyanosis, and syncope [8]. A grade III-VI/VI systolic murmur is typically present, with the point of maximum intensity located over either the left or the right hemithorax [6, 8]. Forty four percent of humans with this condition are reportedly asymptomatic, while fatigue was present in 35%, exertional dyspnea in 17%, cyanosis in 12%, and palpitations in 10% of the cases [17].

## Case presentation

A 1.2 year-old, 28 kg, male intact Golden Retriever, was referred to our Cardiology Service for ascites evaluation. History included lethargy, anorexia and exercise intolerance over the previous five days. Prior to the onset of these signs, the dog was viable and readily able to exercise.

Physical examination showed a severely dyspneic, depressed, dehydrated dog, with moist but pale mucous membranes and a body condition score of 3/9. Rectal temperature was 38.9 °C. The femoral arterial pulse was irregularly irregular and weak, and a right cranial precordial thrill was palpable. Heart auscultation identified a grade V/VI systolic murmur with the point of maximal intensity located ventrad to the right heart-base region. Jugular venous distention was also noted and ascites was confirmed. Respiratory sounds were normal over all lung lobes, while the respiratory rate was elevated at 60 rpm.

**Fig. 1:**
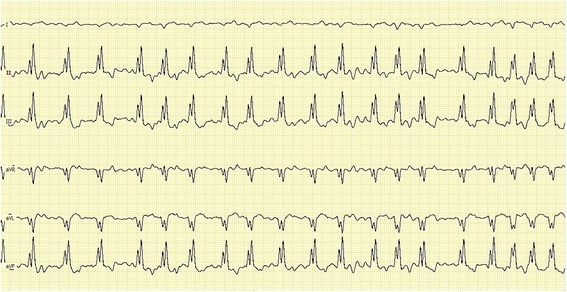
A six-lead ECG strip from a 1.2-year-old male Golden Retriever demonstrating atrial fibrillation with widened (80 ms) and splintered QRS complexes, compatible with a RBBB pattern and mild right axis deviation of the ventricular depolarization mean electrical axis (1 cm/mV, 50 mm/s). Note that the QRS complex, while of small amplitude, is negative in Lead I

An electrocardiographic (ECG) rhythm strip identified atrial fibrillation (AF) with widened (80 milliseconds) and splintered QRS complexes, and a right bundle branch block (RBBB) pattern, with an average ventricular response rate of 190 beats/min (Fig. 1).

**Fig. 2:**
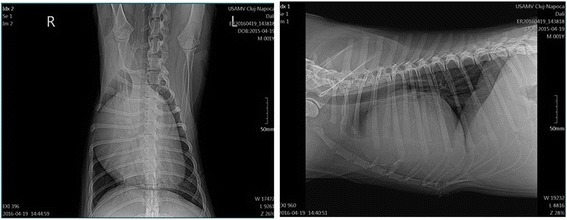
Two radiographs from a male, 1.2-year-old Golden Retriever dog, revealing well-ventilated lung lobes, and severe, right-sided cardiomegaly (vertebral heart sum is 13.3v)

Radiographs revealed well-ventilated lung fields and severe, right-sided cardiomegaly. Vertebral heart score was severely increased at 13.3v (Fig. 2) (reference interval < 10 ± 0.5).

**Fig. 3:**
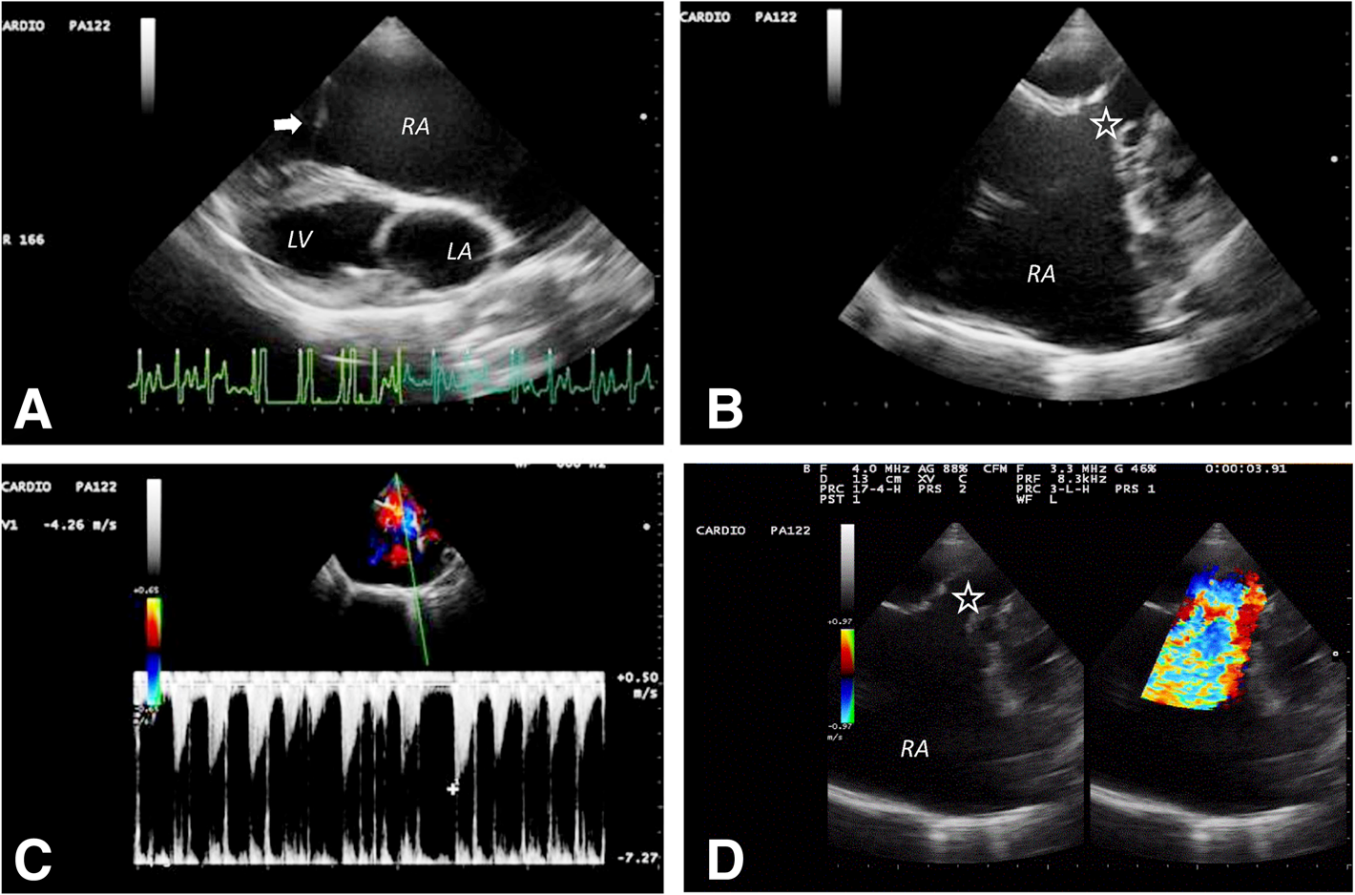
Echocardiograms from a male, 1.2-year-old Golden Retriever dog with atrial fibrillation. **a** A systolic, right parasternal, long axis view demonstrating a severely enlarged right atrium with malpositioned (apically displaced) tricuspid valve leaflet insertions (arrow). **b** A modified left parasternal apical four-chamber view demonstrating incomplete coaptation of the tricuspid valve leaflets (asterisk). **c** A continuous wave Doppler signal from the left apical four-chamber view, demonstrating tricuspid insufficiency (TI) with a peak flow velocity of up to 4.26 m/s in many of the irregularly dispersed cardiac cycles. This velocity is compatible with a systolic pressure gradient of 72.6 mmHg between one of the right ventricular compartments and the right atrium. Note the “V-wave cut off” of the TI signal, suggestive of an elevated pressure in the receiving compartment. **d** A modified left parasternal apical four-chamber view depicting color Doppler evidence of severe TI; LA, left atrium; LV, left ventricle. RA, right atrium

**Fig. 4:**
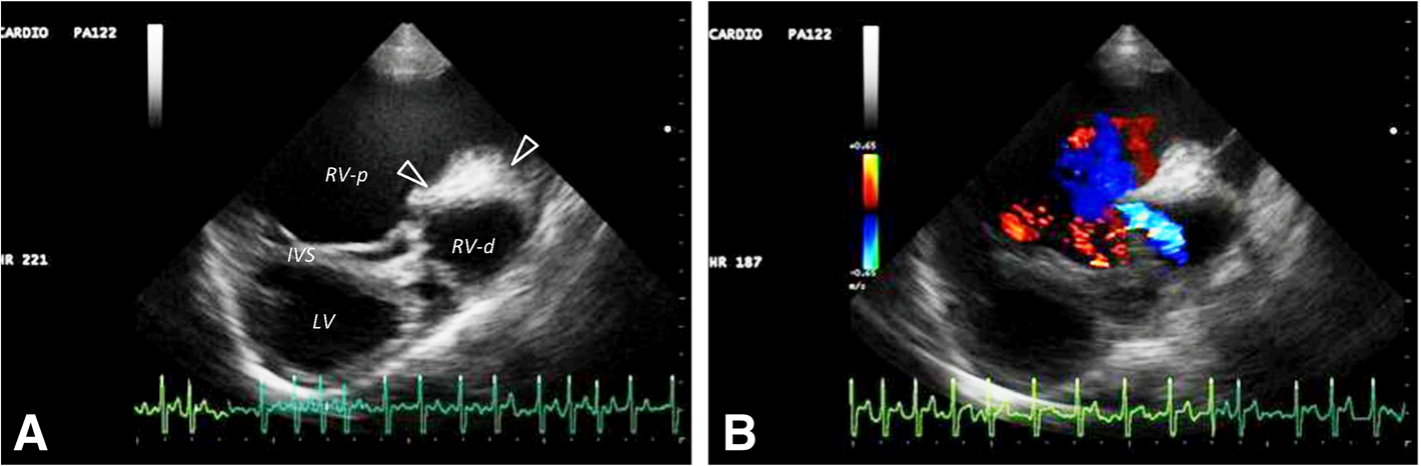
Two-dimensional echocardiograms from the right parasternal short axis view of a male, 1.2-year-old Golden Retriever dog with atrial fibrillation at the level of the left ventricular papillary muscles. **a** There is systolic flattening of the interventricular septum along with a fibromuscular band (arrowhead) dividing the RV into two separate compartments. **b** Color flow Doppler imaging demonstrates a turbulent jet between the two right ventricular chambers. IVS, interventricular septum; RV–d, right ventricular distal chamber; RV–p, right ventricular–proximal chamber

Two-dimensional echocardiography demonstrated a severely enlarged right atrium. There was evidence of congenital TVD: the tricuspid leaflets were malpositioned and displaced toward the right ventricular apex (Fig. 3a). A severe tricuspid insufficiency (TI) jet (Fig. 3d) with a 4.26 m/s peak flow velocity (compatible with a systolic pressure gradient of at least 72.6 mmHg across this valve) was recorded (Fig. 3c). The septal tricuspid leaflet was thickened and attached to very short chordae tendineae; the posterior tricuspid leaflet was also thickened. The right parasternal short axis view, at the level of the left ventricular papillary muscles, demonstrated systolic flattening of the interventricular septum (IVS), attributed to an elevated systolic right ventricular pressure (Fig. 4a). In that same view, an anomalous fibromuscular structure appeared to partially separate the right ventricular cavity into two compartments. A small, systolic, turbulent jet was identified between the two right-ventricular chambers (Fig. 4b).

Spectral Doppler interrogation of the abnormal jet was attempted but accurate velocity measurements could not be performed due to less–than–ideal angulation. However, based on the turbulent appearance of the color flow jet its velocity seemed subjectively high.

The left cardiac chambers and valves demonstrated normal structure and function, and only mild left ventricular (LV) hypertrophy was seen. The left ventricular free wall thickness in end diastole (LVFWd) was 9.9 mm (reference ranges 8.04–8.94 mm), and the interventricular septum thickness in end diastole (IVSd) was 12.08 mm (reference ranges 9.99–11.05) [18].

Pulmonary arterial flow had a normal profile and peak flow velocity. Aortic flow velocity was not accurately measured due to less-than-optimal beam-to-flow alignment.

Serum biochemistry showed only mild liver enzyme elevation. Alanine transaminase (ALT) was 93 U/l (upper reference limit <40 U/l) and aspartate aminotransferase (AST) was 88 U/l (upper reference limit <40 U/l). Hematology was normal.

Five liters of a sero–sanguinotic fluid were removed from the abdominal cavity via abdominocentesis. The fluid contained a low protein (< 2.5 g/dL) concentration, containing only a few scattered cells, including mostly red blood cells, neutrophils, and desquamated mesothelial cells.

The dog unfortunately responded poorly to pimobendan (0.25 mg/kg q 12 h) and furosemide (2 mg/kg q 12 h), and went on to die suddenly four days later.

**Fig. 5:**
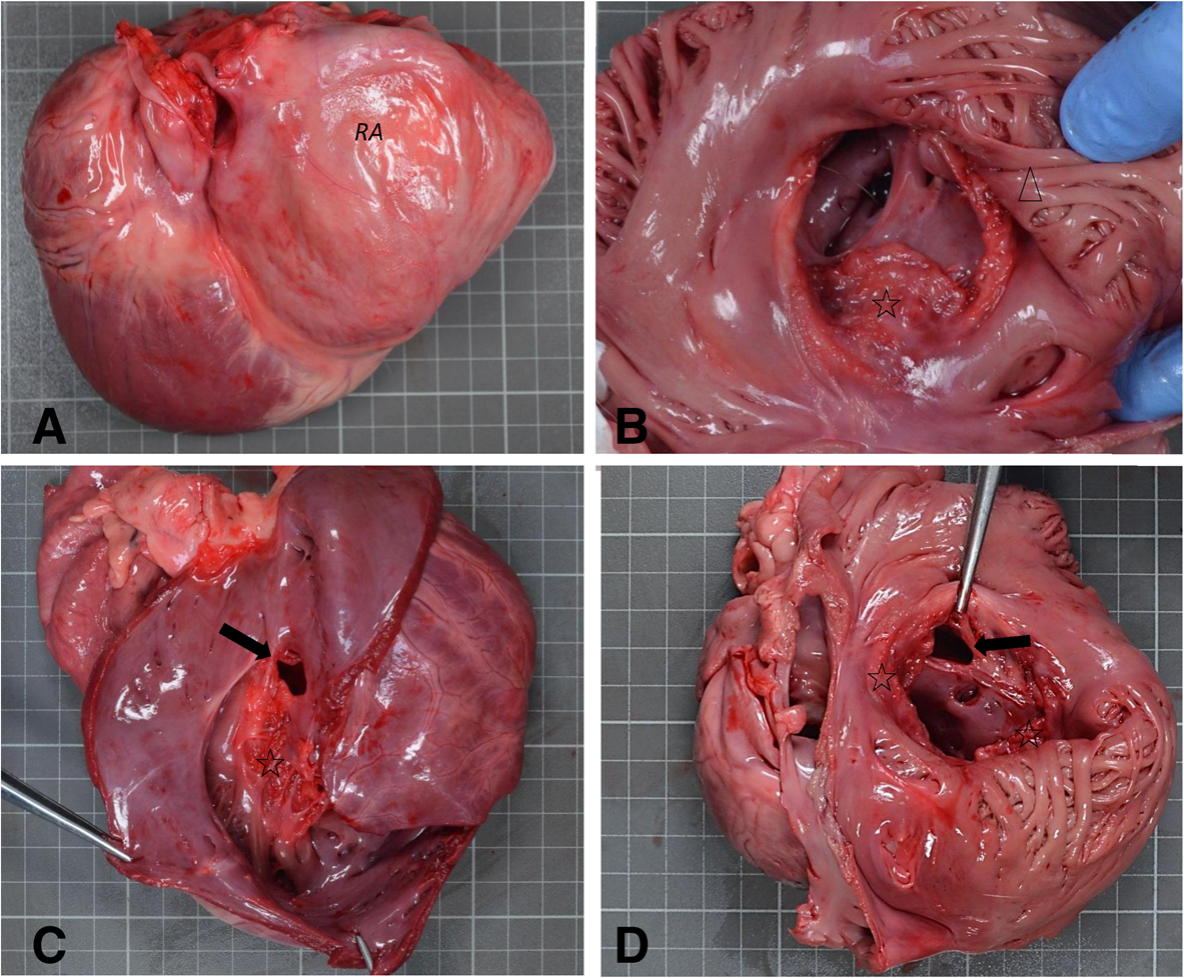
Necropsy findings from a male, 1.2-year-old Golden Retriever dog. **a** The right lateral exterior aspect of the heart, demonstrating a severely enlarged right atrium. **b** The tricuspid valve apparatus seen from the opened right atrium, demonstrating a thickened septal leaflet (asterisk). An arrowhead shows pectinate muscles. **c** A large fibromuscular band (asterisk) seen from the distal RV chamber, divides the RV cavity and includes an oval ostium at its dorsal aspect, communicating between this and the other, proximal RV compartment (arrow). The ostium is seen from the compartment that contains the outflow tract structures. **d** The communicating ostium between the two RV compartments (arrow) seen from the RA, through the compartment that contains the inflow structures, including the tricuspid annulus and leaflets (asterisks). An arrowhead shows right atrial pectinate muscles. LA, left atrium; LV, left ventricle; RA, right atrium; RV–d, right ventricular distal chamber. Each small square represents 1 cm × 1 cm

**Fig. 6:**
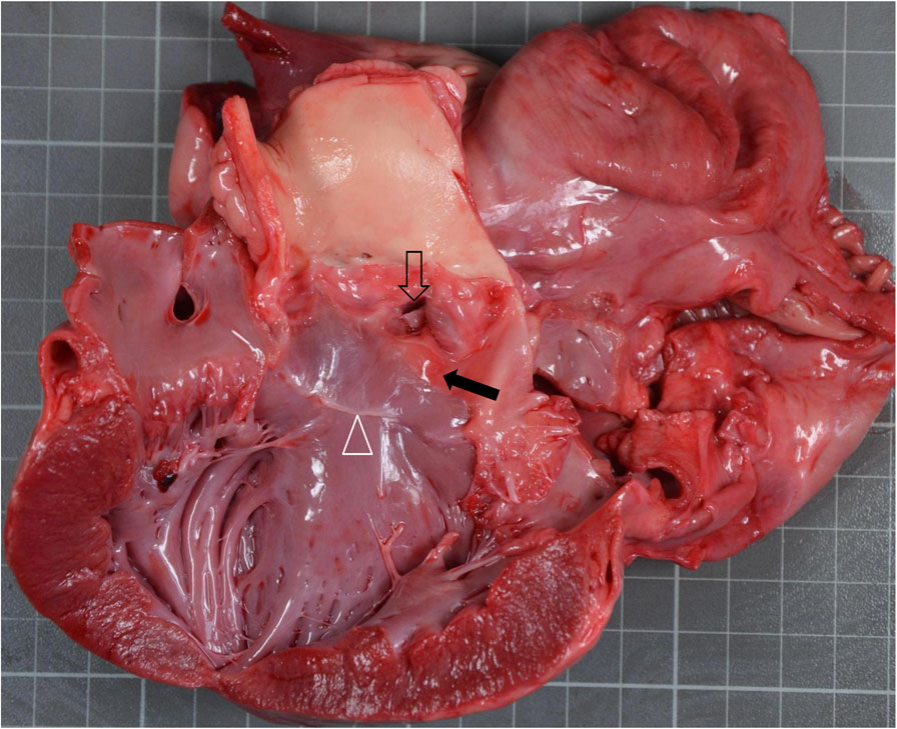
Necropsy findings from a male, 1.2 year-old Golden Retriever dog, demonstrating the left ventricular outflow tract (LVOT) with a small, 4.0 mm wide, perimembranous VSD (transparent arrow) and a small sub-valvar stenotic nodule (black arrow). A fibrotic ring also encircles the LVOT (arrowhead). Mild concentric hypertrophy of the left ventricle (asterisks) is suspected. Ao, aorta; LA, left atrium; LV, left ventricle; RA, right atrium. Each small square represents 1 cm × 1 cm

**Fig. 7:**
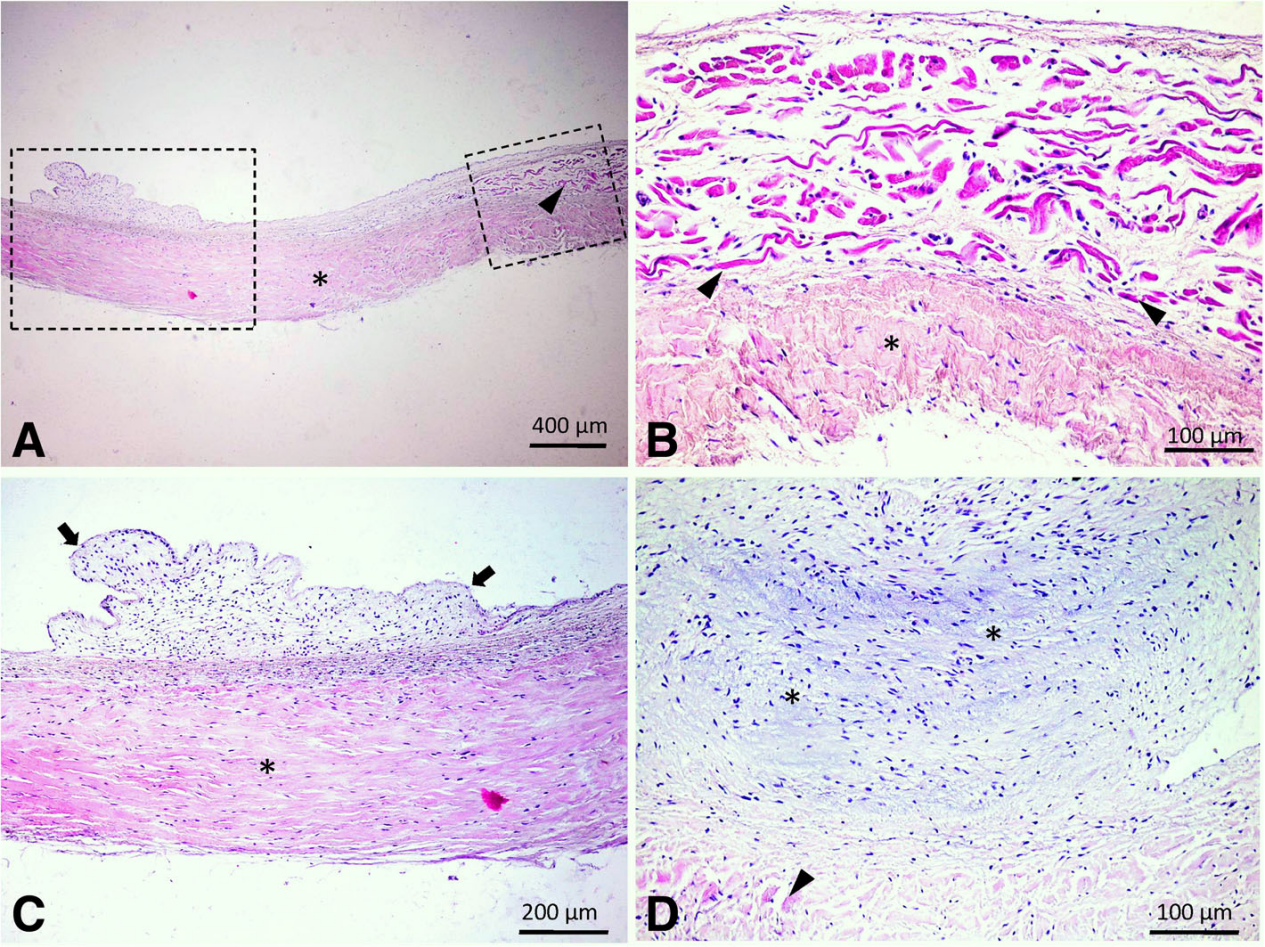
Histopathological features of the fibromuscular band. **a** General view of the structure composed by dense, longitudinally oriented fibrous connective tissue (indicated by an asterisk) and myocardium (indicated by an arrowhead) located mostly at the ventral zone (close to the apex). The endocardium presents areas of nodular hyperplasia. **b** Detail of the area demarcated by the small rectangle in image 7A, presenting the plexiform pattern of the myocardium (indicated by the arrowheads) and the adjacent densely packed fibrous connective tissue (indicated by an asterisk). **c** Detail of the area demarcated by the large rectangle in image 7A. The fibromuscular band shows areas of hyperplasia, marked by sparsely cellular nodules of loose myxoid connective tissue covered by hyperplastic endocardium. **d** A myxoid nodule sharply demarcated from the surrounding myocardium (indicated by the arrowhead), composed of abundant, basophilic extracellular matrix and loosely arranged fibroblasts. H&E stain, ob. X 4 for image A, ×20 for images B and D, and ob.× 10 for image C

Necropsy revealed a severely enlarged right atrium (Fig. 5a). The septal and posterior leaflets of the tricuspid valve were both severely thickened. The septal leaflet was also adhered to the IVS (Fig. 5b). The tricuspid valve annulus was displaced ventrad and caudad, towards the right ventricular apex. The RV was partially divided into two separate compartments by a fibromuscular structure, which was more muscular near the RV apex and more fibrotic near the atrioventricular junction. The structure appeared to be part of the anterior tricuspid valve leaflet, without involving any of the papillary muscles. This impression was supported by the fact that the chordae tendineae of this specific leaflet were of normal length but their insertions were embedded inside the abnormal fibromuscular structure. This fibromuscular structure was attached to the RV apex, at an area rich with coarse septomarginal trabeculation. The two right ventricular compartments communicated only at the uppermost part of the abnormal fibromuscular structure (Fig. 5c, d). The diameters of the oval communicating ostium were 0.8 X 1.2 cm. One right ventricular compartment contained inflow tract components, while the other contained outflow components. A resistive perimembranous VSD measuring 4 mm in diameter was also detected on necropsy, below the aortic valve cusps. The VSD opened into the RV distal chamber. Beneath the aortic valve cusps, there was a small, proliferative, stenotic nodule, partially obstructing the left ventricular outflow tract (LVOT). A fibrotic ring encircled the LVOT as well, at a distance of 1.5 cm from the aortic valve cusps (Figs. 6 and 7). These two combined findings were compatible with mild SAS, which presumably had triggered systolic left ventricular (LV) pressure elevation, based on the mildly pre-mortem, echocardiographically increased LV wall thickness.

Histologically, the fibromuscular band that separates the right ventricle consists of longitudinal layers of dense fibrous connective tissue admixed with cardiomyocytes organized in a plexiform pattern (Fig. 7a, b). The muscular component was present mainly in the ventral area of the band, being largely absent in the central zone. Superficially, the endocardium presented areas of nodular hyperplasia covering mainly the fibrous tissue-rich part of the band (Fig. 7c). The nodules were sharply demarcated and composed of loose connective tissue with myxoid appearance covered by hyperplastic endocardium (Fig. 7d).

## Discussion and conclusions

To the best of the authors’ knowledge, this is the first report of a patient with TVD and DCRV where the anterior tricuspid leaflet is part of an anomalous structure dividing the RV chamber in two compartments that communicated through a single ostium, situated in the dorsal part of the anomalous structure.

As there is no reason to expect that pulmonary hypertension was present, it is likely that one of the two RV compartments was highly pressurized in systole, which would explain both the high-velocity TI jet and the systolic interventricular IVS flattening towards the LV.

In the present case, VSD was restrictive and perimembranous, located just below the aortic valve cusps [19]. In both humans and dogs with DCRV, a concurrent VSD often opens into the more highly pressurized RV (proximal) chamber [11]. However, in the present case, the VSD actually opened into the more distal and lowly pressurized chamber.

The anatomical nature of the TVD in the present case can be thought of as reminiscent of the so-called Ebstein’s anomaly in human beings [20]. In terms of intra-cavitary pressure, Ebstein’s anomaly is characterized by “atrialization” of the dorsal aspect of the RV, due to a downward displacement of the tricuspid septal and lateral leaflet insertions, while the anterior leaflet seems enlarged and appears to be “sail-like” [21]. An autosomal dominant mode of inheritance was proposed in Labrador Retrievers [21] and an autosomal recessive mode of inheritance was suggested in the Dogue de Bordeaux [22]. In the present case, the tricuspid valve septal and lateral leaflets were extremely thickened with restricted motion. The septo-anterior leaflet was prominent, being part of the fibromuscular band dividing the RV. The chordae tendineae of this leaflet were longer than normal, and were attached to the fibromuscular structure.

As the dog presented with clinical signs and physical examination findings of right-sided congestive heart failure (R-CHF), right atrial pressure was likely higher than 15 mmHg. This possibility is also subjectively supported by the “V-wave cutoff” appearance of the spectral Doppler TI-jet morphology (Fig. 3c). When added to the calculated 72.6 mmHg systolic pressure gradient across the tricuspid valve, this translates into an extremely elevated systolic pressure of at least 87.6 mmHg in the (proximal) highly pressurized RV compartment. In fact, the presence of AF suggests that the measured TI systolic pressure gradient was likely an underestimation of the true systolic pressure within this highly pressurized proximal RV compartment, which would suggest that the true systolic pressure within this compartment would have been much higher than 87.6 mmHg, while still in normal sinus rhythm. In addition, this patient’s condition had been severe but also stable for a long enough period, to allow for the development of a severely enlarged right atrial diameter, which eventually contributed to the development of AF. This, in turn, must have exacerbated the hemodynamic compromise and ultimately hastened the onset and progression of R-CHF with not only ascites but also resultant clinical deterioration, to which the patient finally succumbed.

A fourth congenital anomaly observed in this case was a mild SAS, consisting of a fibrous band located 1.5 cm below the aortic valve cusps, and a proliferative nodule situated just below the VSD. In human beings, SAS is commonly associated with DCRV, [3, 4, 23].

To the best of the author’s knowledge, this is the first veterinary case report of a combination of congenital TVD, DCRV, VSD and SAS, where a tricuspid valve leaflet is an integral anatomical component of the fibromuscular anomalous structure dividing the RV into two separate compartments.

## Abbreviations

AF: Atrial fibrillation
ALT: Alanine transaminase
ASD: Atrial septal defect
AST: Aspartate aminotransferase
DCRV: Double chambered right ventricle
ECG: Electrocardiography
IVS: Interventricular septum
IVSd: Interventricular septum thickness in end diastole
LA: Left atrium
LV: Left ventricle
LVFWd: Left ventricular free wall thickness in end diastole
LVOT: Left ventricular outflow tract
PDA: Patent ductus arteriosus
PS: Pulmonic stenosis
RA: Right atrium
RBBB: Right bundle branch block
R-CHF: Right sided congestive right failure
RV: Right ventricle
RV–d: Right ventricular distal chamber
RV–p: Right ventricular proximal chamber
SAS: Subaortic stenosis
TI: Tricuspid insufficiency
TVD: Tricuspid valve dysplasia
VSD: Ventricular septal defect
